# Dr. Clayton L. Hill

**Published:** 1905-12

**Authors:** 


Dr. Clayton L. Hill, of Buffalo, died suddenly of heart disease, at his home, December 25, 1905, aged 66 years. Dr. Hill graduated from the Medical Department of the University of Buffalo in 1864, and immediately began his professional work in this city, in which he continued until his death. He is survived by two daughters, Miss Evelyn Hill of Lafayette, N. Y., Miss Florence Hill of Buffalo, and a son, Robert C. Hill, also of Lafayette.

